# Ecological countermeasures to prevent pathogen spillover and subsequent pandemics

**DOI:** 10.1038/s41467-024-46151-9

**Published:** 2024-03-26

**Authors:** Raina K. Plowright, Aliyu N. Ahmed, Tim Coulson, Thomas W. Crowther, Imran Ejotre, Christina L. Faust, Winifred F. Frick, Peter J. Hudson, Tigga Kingston, P. O. Nameer, M. Teague O’Mara, Alison J. Peel, Hugh Possingham, Orly Razgour, DeeAnn M. Reeder, Manuel Ruiz-Aravena, Nancy B. Simmons, Prashanth N. Srinivas, Gary M. Tabor, Iroro Tanshi, Ian G. Thompson, Abi T. Vanak, Neil M. Vora, Charley E. Willison, Annika T. H. Keeley

**Affiliations:** 1 Department of Public and Ecosystem Health, Cornell University, Ithaca, NY 14853 USA; 2 Medical Research Council Unit The Gambia, London School of Hygiene and Tropical Medicine, London, WC1E 7HT UK; 3 Department of Biology, University of Oxford, Oxford, OX1 3SZ UK; 4 Department of Environmental Systems Science, ETH Zürich, Zürich, 8092 Switzerland; 5 Department of Biology, Muni University, P.O. Box 725, Arua, Uganda; 6 School of Biodiversity, One Health and Veterinary Medicine, University of Glasgow, Glasgow, G12 8QQ UK; 7 Bat Conservation International, Austin, TX 78746 USA; 8 Department of Ecology and Evolutionary Biology, University of California, Santa Cruz, CA 95064 USA; 9 Centre for Infectious Disease Dynamics, Pennsylvania State University, State College, PA 16801 USA; 10 Department of Biological Sciences, Texas Tech University, Lubbock, TX 79409-3131 USA; 11 College of Climate Change and Environmental Science, Kerala Agricultural University, Kerala, 680 656 India; 12 Centre for Planetary Health and Food Security, Griffith University, Nathan, QLD 4111 Australia; 13 School of Biological Sciences, University of Queensland, Brisbane, QLD 4072 Australia; 14 Biosciences, University of Exeter, Exeter, EX4 4PS UK; 15 Department of Biology, Bucknell University, Lewisburg, PA 17937 USA; 16 Department of Mammalogy, Division of Vertebrate Zoology, American Museum of Natural History, New York City, NY 10024 USA; 17 Institute of Public Health, Bengaluru, Karnataka 560070 India; 18 Center for Large Landscape Conservation, Bozeman, MT 59771 USA; 19 Department of Biology, University of Washington, Seattle, WA 98195 USA; 20 Small Mammal Conservation Organization, Benin City, 300251 Nigeria; 21 Department of Animal and Environmental Biology, University of Benin, Benin City, 300000 Nigeria; 22 Australian Capital Territory, Canberra, 2605 Australia; 23 Centre for Policy Design, Ashoka Trust for Research in Ecology and the Environment, Bengaluru, Karnataka 560064 India; 24 School of Life Sciences, University of KwaZulu-Natal, Durban, 4041 South Africa; 25 Conservation International, Arlington, VA 22202 USA; 26 Present Address: Department of Wildlife, Fisheries and Aquaculture, Mississippi State University, Starkville, USA

**Keywords:** Epidemiology, Ecology, Policy and public health in microbiology, Viral infection

## Abstract

Substantial global attention is focused on how to reduce the risk of future pandemics. Reducing this risk requires investment in prevention, preparedness, and response. Although preparedness and response have received significant focus, prevention, especially the prevention of zoonotic spillover, remains largely absent from global conversations. This oversight is due in part to the lack of a clear definition of prevention and lack of guidance on how to achieve it. To address this gap, we elucidate the mechanisms linking environmental change and zoonotic spillover using spillover of viruses from bats as a case study. We identify ecological interventions that can disrupt these spillover mechanisms and propose policy frameworks for their implementation. Recognizing that pandemics originate in ecological systems, we advocate for integrating ecological approaches alongside biomedical approaches in a comprehensive and balanced pandemic prevention strategy.

## Abstract (web summary)

In this Perspective, the authors discuss the importance of preventing zoonotic spillover to prevent pandemics. They highlight mechanisms by which environmental changes can enable spillover, identify ecological interventions for spillover prevention and suggest policy frameworks through which interventions can be implemented.

## Introduction

**Fig. 1:**
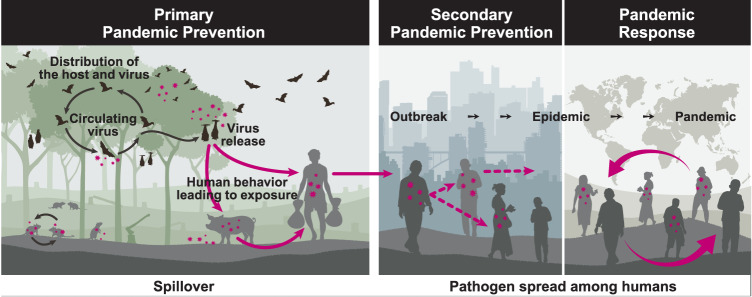
Primary pandemic prevention, secondary pandemic prevention, and pandemic response. Primary pandemic prevention is the set of actions taken to reduce the risk of pathogen spillover from animals to humans, focusing on processes upstream of the spillover event (left panel). By contrast, secondary pandemic prevention (middle panel) focuses on limiting the spread of an outbreak to prevent its escalation into an epidemic or a pandemic. Pandemic response (right panel) involves actions taken to address a pandemic once one is underway. Although not illustrated here, pandemic preparedness involves developing capabilities to respond to a pandemic if one were to occur, and can be implemented concurrently with primary and secondary pandemic prevention. The nature of interventions varies across these phases: Primary pandemic prevention emphasizes ecological and behavioral interventions, but also encompasses biosafety practices in virological research^83^, whereas secondary pandemic prevention and response prioritize epidemiological and biomedical interventions. Definitions: an outbreak is “an increase, often sudden, in the number of cases of a disease in a particular area^84^”; an epidemic is an outbreak extending over a wider geographic area^84^; and a pandemic is “an epidemic occurring worldwide, or over a very wide area, crossing international boundaries and usually affecting a large number of people^84^”.

Reducing the risk of future pandemics requires investment in prevention, preparedness, and response. At present, most attention and funding is allocated to mitigation after a pathogen is already circulating in humans, prioritizing outbreak detection and medical countermeasures such as vaccines and therapeutics^1^. By contrast, primary pandemic prevention—defined as reducing the likelihood a pathogen transmits from its animal host into humans (zoonotic spillover; Fig. 1)^2^—has received less attention in global conversations, policy guidance, and practice^1,2^. Given the time delays in identifying and responding to outbreaks, and the inequity in treatment distributions, investing in pandemic prevention is essential to achieve efficient, equitable, and cost-effective protection from disease.

To effectively prevent pandemics, we must recognize two key points: first that pandemics almost always start with a microbe infecting a wild animal in a natural environment and second that human-caused land-use change often triggers the events–whether through wildlife trade or other distal activities–that facilitate spillover of microbes from wild animals to humans^3^. As land-use change becomes more intense and extensive, the risk of zoonotic spillovers, and subsequent epidemics and pandemics, will increase. Designing land management and conservation strategies to explicitly limit spillover is central to meeting the challenge of pandemic prevention at a global scale.

Herein, we present a roadmap for reducing pathogen transmission from wildlife to humans and other animals. We show how strategic conservation and restoration of nature for reservoir hosts, and mitigation of risks for humans most at risk—what we define as ecological countermeasures—can prevent spillover and protect human and animal health, while also addressing key drivers of climate change and biodiversity loss.

## Mechanisms of spillover

Despite hundreds of thousands of potentially zoonotic microbes circulating in nature^4^, pandemics are rare. Microbes, termed pathogens if they cause disease, must overcome a series of barriers, simplified and described below, to transmit from a wild animal to a human. Crossing those barriers requires the alignment of specific conditions—including ecological, epidemiological, immunological, and behavioral conditions—that are often complex and dynamic^5^.

First, the distribution of the species that maintains the zoonotic pathogen in nature (the reservoir host) and the species that is infected (the recipient host) must be connected, usually through overlapping distributions. Once wildlife reservoir hosts and humans overlap, the second barrier is the immune functions within wildlife hosts that keep potential zoonotic pathogens at low levels. Particular stressors (e.g., habitat loss, lack of food) can increase host viral infection and shedding^6^. A pathogen that passes through this second barrier and is shed by the animal host encounters a third barrier: humans must be exposed to a pathogen for spillover to occur. That exposure depends on specific interactions or behaviors of humans and the virus-shedding host. Exposure to the pathogen may be through direct contact, such as a bite, or indirect contact with the reservoir host’s excreta or a non-vertebrate vector (e.g., blood-feeding parasite). Often a bridging host species, such as commercially traded wildlife or a domestic animal, is infected by the reservoir host and subsequently amplifies and transmits the pathogen to humans. The fourth barrier is human susceptibility. The pathogen must be able to establish an infection within humans by overcoming structural and immunological barriers (e.g., binding to a human cell). Those barriers are substantial–one reason pandemics are rare–protecting humans from a continuous rain of microbes from soils, plants, and animals^5^. Fifth, after establishing an infection within a single human, the pathogen must be able to amplify within this new host, be excreted (e.g., through respiration), and then transmitted onward and exponentially^7^. If any of these barriers is not overcome, a pandemic cannot occur^5^.

### Land use-induced spillover

**Fig. 2:**
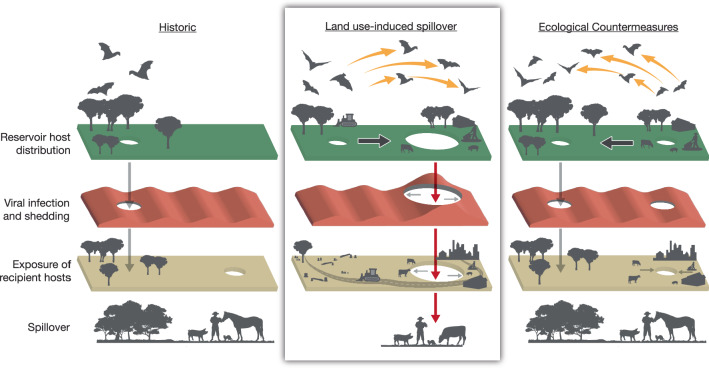
Land use-induced spillover and ecological countermeasures. Historic (left panel): Historically, reservoir hosts and large human populations (and their domestic animals) were more separated, viruses circulated at low levels with seasonal fluctuations in prevalence, and the holes in the barriers to spillover were small and did not align^5^. Land use-induced spillover (middle panel): Land-use change increases the risk of spillover by driving two phenotypic changes in reservoir hosts: changes in behavior that alter how they use space, and changes in reservoir host energy and stress levels (allostatic load) that influence viral infection and shedding. Land-use change can also lead to emergent human behaviors that increase exposure to pathogens. Land-use change generally increases the overlap of reservoir, human, and bridging hosts; increases the probability that reservoir hosts are shedding pathogens; and increases the probability that humans are exposed to those pathogens. In sum, these changes increase the size and alignment of the holes in the barrier to spillover. Ecological countermeasures (right panel): Ecological countermeasures can address all three issues. Retaining natural resources reduces the overlap of humans and domestic recipient hosts in space and time, reduces the probability of allostatic overload and reduces the likelihood of emergent human behaviors that facilitate exposure.

Intact ecosystems provide the first line of defense against new pandemics because they strengthen the first three barriers to spillover (minimizing distribution overlap, host stress, and human exposure) and hence decrease the likelihood that the conditions for spillover occur or align^3^. Conversely, land-use changes and other environmental disturbances erode those first three barriers to spillover by changing the reservoir hosts’ spatial behavior and allostatic load (energy and stress budget), as well as altering human behavior. In this context, we identify targeted ecological countermeasures designed to decrease these risks (Fig. 2).

We focus on ecological countermeasures in bats since several major epidemics and pandemics (e.g., those caused by SARS-CoV-2, Ebola virus, SARS-CoV-1, MERS-CoV, and Nipah virus) have an evolutionary origin in bats (but notably do not cause disease in their bat reservoir hosts)^8^. Certain bat species are also the hosts of four of the nine diseases prioritized by the World Health Organization as having the potential to generate epidemics that pose a great risk to public health, and for which there are insufficient countermeasures^9^. However, the ecological countermeasures we present also apply to other host taxa, particularly species that are susceptible to local resource depletion and can sustain the circulation of potential pathogens (e.g., species that aggregate in large numbers like colonial nesting birds, or in spatially structured but extensive aggregations, such as prairie dogs and other rodents). For species tied to permanent refuges (roosts, breeding grounds, burrow systems and warrens), loss of habitat may quickly push populations into allostatic overload or in more mobile species, prompt resource tracking and migration with attendant energetic costs and risks.

### Reservoir host energy and stress (allostatic load)

**Fig. 3:**
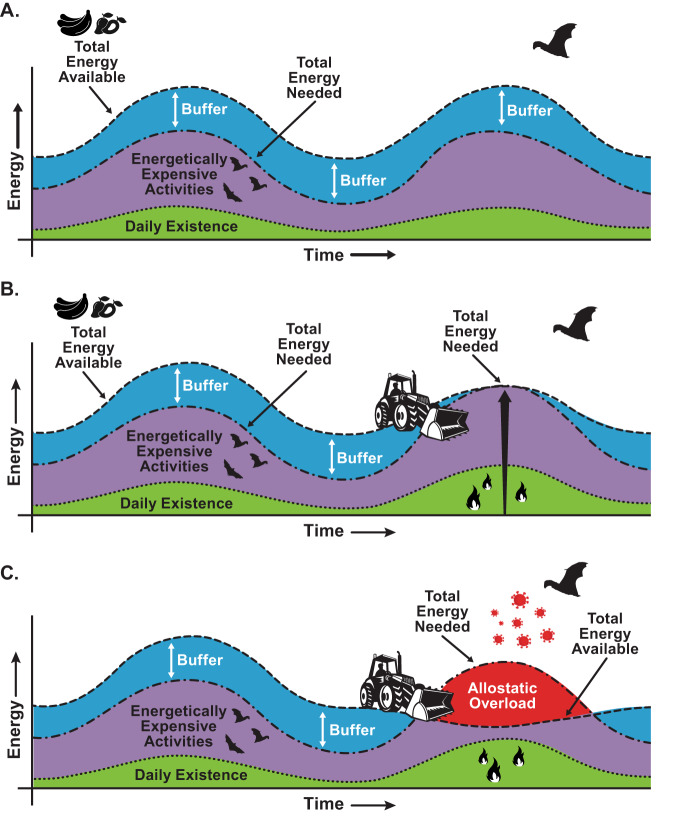
Allostatic overload as a key driver of pathogen spillover. Bats have evolved mechanisms to meet their exceptionally high energy needs under prevailing environmental conditions. **A** Baseline levels of energy (green) are required for basic daily activities – to fuel cells, to move around, to find food and water, and to maintain the immune system. At any given time, a certain amount of food - or energy - is available (blue+purple+green), which varies seasonally. Bats optimize their energy intake and energy expenditure, timing expensive activities like migration and reproduction (purple) to periods in which more food is available. Under normal conditions, an energetic buffer (blue) exists providing energetic wiggle room for years with poor food availability. **B** Perturbations in the environment, whether natural (e.g., fire in some instances) or man-made (e.g., downstream effects of global climate change, habitat destruction, etc.) increase the amount of energy needed for survival and reproduction. For example, animals may be required to travel greater distances to locate food and resting sites. Such increased exertion diminishes the energetic buffer that enables them to withstand periods of resource scarcity. **C** At its worst, these perturbations result in a reversal of fortune; less energy is available than the bat needs. In these conditions, or with disturbance or harassment, animals experience allostatic overload (red). This leads to suppression of immune function, and increased susceptibility to viral infection and shedding. Figure adapted, in part, from concepts in^10^.

Healthy animals maintain a positive energy balance, where energy inputs either from foraging or stored reserves of fat, balance or exceed energy expenditure required for survival and reproduction (Fig. 3). This balance of energy in physiological systems occurs through allostasis—a dynamic process that integrates the neuroendocrine, metabolic, cardiovascular, and immune systems to adapt to varying conditions. Animals regularly adapt to increased energy demands needed to migrate, hibernate, or reproduce. The total resources an animal requires at any given time is an animal’s “allostatic load”^10,11^. Allostatic load is frequently estimated with biomarkers such as cortisol, a glucocorticoid hormone indicative of stress^12^, or related energetic and immune metrics, such as total white-blood-cell count, the neutrophil-to-lymphocyte ratio, and immune regulatory markers. When in balance, glucocorticoid hormones help manage energy usage and have generally beneficial effects on immunity. For example, they mediate anti-inflammatory processes, support T cell maintenance, and enhance the functions of Th2, Th17 and B cells, which collectively bolster the body’s defense against infection and keep immune responses in check^13,14^. Across millennia, animals evolved the capacity to maintain allostasis under predictable variations in their environments, precisely aligning energetically expensive activities with periods of maximum food availability^15^ (Fig. 3).

Animals are less able to manage the physiological and behavioral challenges that arise from unpredictable environmental changes, particularly those caused by human activities. Perhaps the most common consequence of environmental change is decreased food availability, leading to weight loss^16^. When food is limited, energy expenditure may exceed energy input and the animal shifts into a state of allostatic overload (Fig. 3).

Habitat destruction, degradation, and fragmentation profoundly increase the likelihood of allostatic overload. This risk is compounded when animals face repeated stressors, such as cave disturbance or harassment^17^. To survive, animals must divert energy from other systems, including their immune defenses^14,16^. The effects of allostatic overload are largely mediated by the chronically elevated glucocorticoid hormones, which can lead to immune system dysregulation, impaired resistance to infection, and a shift in the balance between pro-inflammatory and anti-inflammatory processes. This state, the effects of which accumulate over an animal’s lifetime, facilitates viral infection and shedding^13,18–20^. Consequently, animals experiencing allostatic overload may shed more pathogens for longer periods, increasing the risk of spillover. Empirical evidence underscores the link between stress, acute food deprivation, and low body weight with higher probability, magnitude, and duration of viral shedding, as observed in bats^21–25^ and birds^26,27^.

### Reservoir host spatial behavior

Changes in land use not only affect the energy needs of reservoir hosts but also alter how reservoir hosts use space, including how they encounter humans, livestock, or other bridging hosts. Typically, animals have home ranges sufficient for them to acquire the resources they need such as food, water, shelter, and mates. Some species, especially those dependent on unpredictable or briefly available food, may need to migrate or move regularly to find these resources. Land-use changes can limit the amount and accessibility of food resources. In response, and to avoid or mitigate allostatic overload, animals often need to expand their search area or modify their home ranges to find sufficient food^28,29^. For example, fruit-eating bats *Dermanura watsoni* were observed to have larger daily feeding ranges in degraded habitats^30^. Such adaptations may increase the likelihood of encounters and, consequently, pathogen transmission between reservoir hosts, humans, and livestock. This may be especially true if they must traverse resource-sparse areas to find food, increasing stress and mortality risk. A study in Uganda, for example, showed increased contact between humans and non-human primates with increasing forest fragmentation^31^.

Moreover, wildlife populations may adapt to areas where they historically did not occur, and some species that host zoonotic pathogens have proven more likely to thrive in disturbed landscapes than in undisturbed sites^32^. For example, in response to the loss of winter habitat, Australian *Pteropus alecto* bats, carriers of Hendra virus, are shifting to agricultural and urban areas. Here, they feed on suboptimal but reliable foods in proximity to livestock^33^.

Increased zoonotic risk, then, often coincides with stressful life stages or times and places of resource scarcity^21,33,34^. Understanding which animals are most likely to modify their distributions, or are at the highest risk of allostatic overload, helps target countermeasures to spillover. For example, the *P. alecto* bats that shifted to novel agricultural and urban habitats shed higher levels of Hendra virus than bats in traditional habitats, especially during winter and after periods of food scarcity^22,35^. This combination of factors breaches the barriers earlier noted and has led to a higher probability of spillover^22^.

### Human behavior

Although human interaction with a pathogen is a fundamental component of pathogen spillover, mere spatial overlap between humans and virus-shedding reservoir hosts is not sufficient for spillover. Specific human behaviors (not always within one’s control) that provide a transmission route and sufficient dose for infection are usually required—for example, harvesting guano or date palm sap^36–38^, visiting a tourist cave^34^, or butchering wildlife with inadequate protection^39^. Such behaviors, which increase the frequency and intensity of contact with wildlife and wildlife excreta, can become more prevalent because of land-use change, frequently precipitated by the construction of new roads. While road construction, if designed well, can bring benefits such as employment, reduced transportation costs, and development^40^, roads also facilitate increased access to wildlife habitats. This access can enable activities such as the extraction of wild animals for food and trade, timber harvest, and livestock grazing, following deforestation^41,42^. New settlements that follow roads may also promote synanthropic responses of wildlife; for example, bats are commonly found roosting on roofs of rural homes^43^.

Road construction not only alters exposure opportunities but also introduces people into communities that lack immunity to local pathogens. By contrast, Indigenous Peoples and local communities (IPLCs) who have coexisted with these environments may have some protective immunity to local pathogens through repeated exposures. This is evident from the presence of antibodies to various outbreak-prone viruses in populations with frequent wildlife exposure. For example, antibodies to filoviruses were detected in bat harvesters in remote northeast India^44^ and antibodies to SARS-related coronavirus have been identified in people residing near caves in Yunnan Province, China^45^. Such evidence suggests that while pandemics may be rare, local spillovers could be relatively common. Furthermore, the construction of roads not only increases the risk of exposure for those lacking immunity but also facilitates the rapid spread of novel pathogens once they have entered the human population, thereby increasing the likelihood of a pandemic.

Apart from the direct impact of road construction, there is a multitude of factors relating to deforestation and forest degradation that could affect human exposure to pathogens, including agricultural practices such as the cultivation of palm oil and extractive industries, notably mining^46^. Typically, such activities are either preceded by or necessitate the building of roads, further intertwining human exposure with infrastructural development. IPLCs living in and around forests, aren’t always the main beneficiaries of these activities and can be actively harmed by them^47,48^. For example, land-use change can result in decreased income and food security, incentivizing some individuals to increase hunting and bush travel. This underscores the need for development projects, including road construction, to take holistic approaches that optimize outcomes for people rather than focusing on single outcomes that can have unintended consequences. Such an approach could deliver much of the economic benefits to people while reducing environmental and social damage. Individual human behaviors that increase spillover risk must be considered in the context of such socio-ecological factors–including vulnerabilities and inequalities—as well as in a historical and cultural context^49^.

## Ecological countermeasures defined

We define ecological countermeasures as actions that protect and restore wildlife habitat or mitigate wildlife-human interactions to reduce the risk of pathogen spillover. These measures are strategically designed to increase the resilience of reservoir host populations, reduce stress and likelihood of viral shedding, prevent distributional shifts, and protect vulnerable human communities. By addressing these factors, ecological countermeasures target the root causes of spillover. They effectively strengthen barriers to spillover and decrease the likelihood that the conditions for spillover align.

**Fig. 4:**
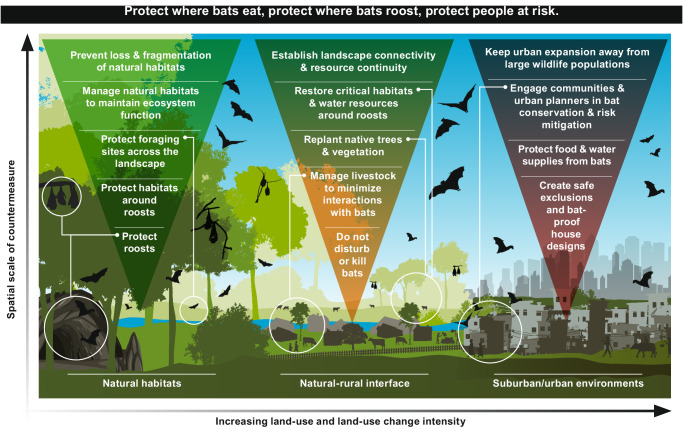
Proposed ecological countermeasures over different spatial scales and land-use intensity gradients. We propose a tiered approach that considers the land-use context surrounding the habitats of reservoir hosts. Because the next pandemic is most likely to be triggered by a pathogen that is currently limited in its exposure to human populations, the highest priority should be to preserve intact ecosystems and enhance their resilience through restoration and increasing connectivity. In regions where humans and reservoir hosts share landscapes, we prioritize the safeguarding of critical areas needed for reservoir hosts’ feeding, resting, and social aggregation. Simultaneously, we aim to protect human communities and livestock most at risk of exposure to zoonotic pathogens.

We propose a tiered approach that considers the land-use context surrounding the habitats of reservoir hosts (Fig. 4), focusing on enhancing habitat integrity, heterogeneity, and connectivity. In our view, the most effective strategy to reduce the probability of another pandemic is to preserve intact ecosystems and bolster their resilience through restoration and the creation of buffer zones. This priority is driven by the likelihood that the next pandemic will be triggered by an as-yet-unknown pathogen, referred to as “Disease X” by the World Health Organization^50^, that has had scarce opportunities for spillover or for evolutionary adaptation in bridging hosts. Our primary emphasis should be on maintaining and enhancing the integrity and resilience of still-intact landscapes to prevent new interfaces that could enable the emergence of Disease X.

In regions where humans and reservoir hosts share landscapes, we prioritize the safeguarding of critical areas needed for reservoir hosts’ feeding, resting, and social aggregation. Simultaneously, we aim to protect human communities most at risk of exposure to zoonotic pathogens. In the following sections, we explain how these strategies target the fundamental drivers of pathogen spillover and promote the health of both wildlife and human populations. While we focus on bats as reservoir hosts, ecological countermeasures are relevant across diverse reservoir host species, as long as specific ecological contexts and local practices are considered^51^. We present these strategies with a simple policy-focused message as they would apply to bats: protect where bats forage (where bats eat), protect where bats roost (where bats sleep), and protect people at risk (Fig. 4).

### Protect where bats forage

The quality of foraging areas determines the energetic buffer protecting individuals from allostatic overload in times of increased energetic costs or reduced resource availability (Fig. 2b). If animals have enough nutritious food, they are less likely to become energetically or physiologically stressed, reducing the risk of allostatic overload and infection and shedding (Fig. 2c). Moreover, the location of bat foraging areas relative to human activity determines the spatial overlap with potential recipient hosts. If enough food is available in relatively unmodified landscapes, or immediately around roosts, bats are also less likely to use areas with higher human population densities. Thus, protecting where bats eat not only ensures that they are healthy, but that they are spatially separated from people.

In natural landscapes (Fig. 4, left panel), the overarching priority is to preserve or improve the integrity of ecosystems that animals inhabit, as previously outlined. This may entail securing extensive areas of unmodified habitats, and proactively managing these landscapes to prevent fragmentation and degradation.

In landscapes that have already been degraded (Fig. 4, middle panel), the focus should shift to protecting, restoring, and connecting key food sources that sustain reservoir hosts during periods of resource scarcity (e.g., winter or the dry season) and through energy-demanding life stages (e.g., pregnancy and lactation). Additionally, in environments facing degradation from land-use and climate change, ecological countermeasures are crucial for mitigating food shortages caused by habitat deterioration across multiple scales.

The natural-rural interface often presents a heterogeneous landscape to bats, characterized by a mix of high-quality foraging habitats embedded in or interdigitating with degraded habitats or areas of human land use. These areas, while fragmented, can still offer valuable nutritional resources. It is crucial to protect key foraging sites, especially those outside of protected areas, and to preserve habitats surrounding roosts. A priority is to maintain or create connectivity among quality habitat patches to ensure a consistent flow of resources. Thereafter, efforts should be directed towards the restoration of critical habitats and water sources, particularly in the vicinity of roosts, coupled with strategic livestock management to reduce interactions with bats. Active management strategies should aim to maximize the benefits of human land-uses such as croplands and plantations, for both humans and bats^52,53^.

In suburban and urban settings (Fig. 4, right panel), priority activities focus on the separation of bats and people through strategic planning and restricting human access. At the broadest scale, urban expansion plans should avoid encroaching on large wildlife habitats. Within urban areas, it is crucial to preserve bat foraging resources without inadvertently increasing contact with human populations. This necessitates a collaborative effort between local communities, urban planners and bat experts who understand the requirements of local species. For example, ornamental or landscaping trees used in city planning may attract fruit-eating bats (such as members of the Pteropodidae and Phyllostomidae families) in subtropical and tropical regions. This is also true for fruit trees in residential backyards^54^. A practical approach might include selecting alternative landscaping species and planting bat-attractive trees in areas that are less accessible to humans. Wildlife-safe protective netting around backyard fruit trees can also limit bats’ access to ripe fruits and minimize fruit loss^43,52,53^. Box 1 provides real-life examples of preserving or enhancing bat foraging habitat and Supplementary Table 1 provides more examples of ecological countermeasures.

Box 1 Real-life examples illustrate the importance of protecting or enhancing where bats forage

- In subtropical Australia, no Hendra virus spillovers occurred when *Pteropus* species bats left agricultural areas to feed on pulses of nectar in winter-flowering forests^33^. In some areas of the subtropics, over 90% of these crucial habitats have been cleared and the remaining forest flowers on multi-year cycles. Consequently, the occurrence of abundant winter flowering has become increasingly rare^33^. Restoring these habitats would target animals’ needs during predictable periods of scarcity, decrease their allostatic load, and reduce their reliance on human-dominated areas for food. Replanting winter habitats would be a sustainable, scalable, and effective strategy to reduce the risk of spillover of not just Hendra virus, but other viruses carried by *Pteropus* species bats.
- Great fruit-eating bats (*Artibeus lituratus*) captured in areas of Colombia that used agroforestry had higher body weights and body condition scores than those within conventional farming areas^85^. Thus, emphasizing agroforestry in agricultural landscapes can provide critical food and shelter for bats^86–90^. In turn, bat predation of agricultural insect pests provides economic and ecological benefits to agriculture by increasing crop yields and reducing pesticide applications^90^.
- To improve the foraging efficiency of wild little brown bats (*Myotis lucifugus*), insect density was increased using UV light lures^91^. This approach aimed to reduce the bats’ allostatic load and their susceptibility to white-nose syndrome, a disease caused by a fungal pathogen that does not pose a risk of spillover to people. Increased fat reserves can improve a bat’s ability to survive this disease. Bats had reduced commuting costs and increasing foraging efficiency, demonstrating that bats behaviorally respond to increased prey availability during critical energetic periods. This work highlights the potential benefits of restoring and enhancing habitats near bat hibernacula to improve the resilience of reservoir host species.
- Agave plants are being restored along bat migration corridors in the southwest United States and northeast Mexico to provide nectar for Mexican Long-nosed bats (*Leptonycteris nivali*) and Lesser Long-nosed bats (*Leptonycteris yerbabuenae*) during energetically expensive migration^92^. In the first five years, over 80,000 agaves were planted within 50 km of six key bat roosts, encompassing both migratory and maternity roosts. This restoration effort not only aids bats but also benefits farmers and rural communities in Mexico, as wild agaves are also harvested for food and beverages, livestock fodder, fencing materials, and other uses. Agaves hold significant cultural value and contribute to the livelihoods of rural Mexican communities^92^. Consequently, restoring bat foraging habitat is an example of how conservation efforts can simultaneously enhance human well-being when co-benefits are identified and integrated.

### Protect where bats roost

Roosts are locations where bats sleep, shelter, mate, socialize, and raise their young. With few exceptions, bats cannot construct shelters and must roost in pre-existing natural (e.g., caves, rock crevices, tree cavities, and tree foliage) or human-made (e.g., buildings, bridges, mines) structures. Moreover, species are typically highly selective of their roost sites, seeking out particular microclimates, light conditions, ingress, and egress conditions. The number of bats using a roost can vary greatly, containing anywhere from a few bats to hundreds of thousands, depending on the species and nature of the roost.

Protecting the roost includes minimizing disturbance and persecution—conversely, often a first response to an outbreak of a bat-borne pathogen. Disturbance not only causes stress, impairing their immune responses but can also force bats into new areas. This increases their energy expenditure and likelihood of contact with humans^22,55^. Moreover, culling bats has been linked to increased active infection within bat populations (e.g., rabies in vampire bats [*Desmodus rotundus*^56^] and Marburg virus in Egyptian fruit bats [*Rousettus aegyptiacus*^21^], and a greater risk of spillover.

Roosts are typically small natural features, and protecting roost sites is a specific management action that can reduce the risk of pathogen spillover. This may require establishing protection buffers around roosts or installing physical barriers (Fig. 4, and Supplementary Table 1). Such buffers are also vital for preserving the quality and quantity of foraging habitats surrounding the roost. Engaging local communities is another key strategy, especially if the roost holds cultural or use value, as is common with caves^57^. Local communities are less likely to harm bats if they are aware of bat natural history, and have previously engaged in environmental education^58^, and are aware of the benefits of bat presence^59^.

### Protect people at risk

The third countermeasure, focused on the safety of humans and livestock in proximity to reservoir hosts, is less ecologically oriented but is crucial in mitigating pathogen exposure risk (Fig. 4, Supplementary Table 1). Pathogen exposure can occur through contact with reservoir hosts, their body fluids, excreta, or through aerosols and droplets derived from these sources. Thus, identifying and modifying human behaviors that elevate the risk of such exposures is essential.

For communities reliant on bat-associated economic activities, such as guano harvesting, tourism, and wildlife consumption^45,56,60,61^, adopting safe practices is critical (Supplementary Table 1). Additional measures may include restricting and regulating the trade of bats^62^ and preventing contact between bats and farmed wildlife^63^. When the specific mechanisms of pathogen spillover are understood, the implementation of preventative measures can be relatively straightforward. In Bangladesh, an effective measure to prevent Nipah virus transmission is covering the areas of date palm trees where sap is collected, which prevents bats from contaminating the sap and transmitting the Nipah virus to humans^64^. In Malaysia, a regulation requiring fruit trees to be planted at a distance from pig sties may explain the lack of subsequent Nipah virus spillovers^65^. Similarly, keeping horses away from trees frequented by bats at night may reduce the risk of Hendra virus transmission between bats and horses^66^.

Box 2 lists interventions in the context of the degree of human landscape modification. Future work must assess the relative effectiveness, feasibility, and prioritization of these countermeasures across different countries and regions since the underlying conditions and legal landscapes will vary. Additionally, given the dynamic nature of climate and land use-induced changes impacting natural and human environments, a flexible, iterative, and adaptive approach is essential for prioritization of these countermeasures^67^.

Box 2 Countermeasures in the context of degree of human landscape modification Ecological countermeasures that protect where bats eat and roost, and protect people at risk, must consider the activities of bats and humans in the landscape. Countermeasures can be implemented at a range of geographic extents and within different contexts of degrees of human modification (Fig. 4 and Supplementary Table 1)

**In large wild areas, protect where bats forage and roost:**

- Maintain or increase the integrity of ecosystems by preventing the destruction and fragmentation of natural areas.
**In shared landscapes dominated by natural areas interspersed with human land uses:**
- Protect where bats eat:
  - Connect protected areas.
  - Preserve and restore vegetation diversity and structural complexity in bat foraging habitats.
  - Protect and restore habitats that provide food during periods of resource scarcity and high energetic demand.
  - Maintain or restore landscape heterogeneity through, for example, wide buffers of natural vegetation along sensitive habitat like streams and wetlands.
  - Promote sustainable agriculture and forestry practices that support bat foraging and roosting.
  - Minimize disruption to water sources used by bats.
  - Protect natural areas when planning new developments.
- Protect where bats roost:
  - Limit human access to roost sites to minimize disturbances.
  - Create buffers of foraging habitat around known roosts.
  - Protect a diversity of roosting options for bats, including large cavity-bearing trees, tree snags, and caves.
  - Provide alternative roosting options such as boxes and hollow trees.
- Protect people at risk:
  - Manage livestock to reduce interactions with bats and bat excreta.
  - Provide information on risks and risk mitigation associated with certain activities.
  - Use personal protective equipment for individuals in contact with bats or their excreta.
  - Vaccinate at-risk populations for endemic bat-borne pathogens such as Ebola or rabies and potentially against pandemic potential pathogens in the future.
  - Empower communities as stewards of the local land and wildlife, including bats.
**In heavily modified landscapes such as intensively farmed and urban areas:**
- Preserve where bats eat and roost:
  - Conserve remaining natural habitats that provide shelter or food.
  - Maintain and restore connectivity.
  - Restore foraging habitat near roosts.
  - Restore habitat buffers around roosts.
  - Increase the proportion of native plant species that provide food and shelter for bats in remnant natural areas away from people.
- Protect people at risk:
  - Exclude bats from human food (e.g. fruit trees) and water supplies.
  - Exclude humans from roosts in public buildings and structures (e.g. churches, bridges, culverts).
  - Humanely exclude bats from houses and construct bat-proof housing.
  - Actively involve communities in risk mitigation measures.

## Policy outlook

Currently, multilateral policy discussions focus predominantly on enhancing pandemic preparedness (e.g., developing new vaccines, readying healthcare systems)^1,68^. While these capacities are undeniably important, integrating a more balanced approach that also prioritizes spillover prevention could reduce human suffering and negative economic impacts in the long term. Despite this, prioritizing prevention proves challenging and is overshadowed by reactive strategies that are activated only after a pathogen is already circulating among humans. This is evident in the current draft of the World Health Organization (WHO) Pandemic Agreement, which does not mention “primary pandemic prevention” and uses the word “prevention” only in the context of secondary prevention measures such as early detection and outbreak response^69^.

Although the importance of pandemic prevention is well-acknowledged, the concept of using ecological countermeasures—actions that protect and restore wildlife habitat or mitigate wildlife-human interactions—as a preventative strategy is only emerging. Ecological countermeasures offer multiple advantages: not only can they prevent spillover, but they engage multiple sectors in action beyond public health, and they contribute multiple co-benefits including climate change mitigation, biodiversity protection, and added ecosystem services (e.g., pest control and pollination by bats). Feedback among these sectors calls for integrated approaches. For example, both climate change and biodiversity loss can intensify processes that drive spillover. Excess heat, extreme climate events, and changing plant phenology are likely to increase allostatic load and alter wildlife (and human) spatial behavior^70^. The loss of biodiversity, including predator species, often leaves ecosystems dominated by species that are more competent hosts for zoonotic pathogens^32^. Together these processes escalate the need for ecological countermeasures.

Ecological countermeasures support, strengthen, and work in accord with existing and future policy frameworks, including those under the United Nations Framework Convention on Climate Change’s Paris Agreement, the Convention on Biological Diversity (CBD)’s Kunming-Montreal Global Biodiversity Framework, the UN Sustainable Development Goals, the UN Decade on Ecosystem Restoration, the new Pandemic Fund through the World Bank, and the WHO Pandemic Agreement. Such existing policy efforts offer opportunities for nations to invest in and incorporate primary pandemic prevention alongside preparedness efforts^1^.

Centrally, ecological countermeasures are fundamentally equitable because health benefits almost always accrue regardless of access to health systems. We’ve seen with COVID-19 and mpox that the most vulnerable populations, at greatest risk of infection and adverse outcomes, often had limited access to vaccines^71^. By contrast, spillover prevention benefits everyone globally, irrespective of individuals’ access to health systems^1,72,73^.

### An Intergovernmental Panel for Pandemics

Many international entities have mandates that include enhancing pandemic prevention, preparedness, and response, including the One Health High-Level Expert Panel, the Global Preparedness Monitoring Board, and the Quadripartite. Such bodies all address unique and important issues, but none acts as an official scientific body that regularly assesses and synthesizes the full breadth of the latest data on pandemic prevention, preparedness, and response.

To address this, we strongly support the establishment of an Intergovernmental Panel for Pandemics, which could eventually come to fruition with the passage of the WHO Pandemic Agreement. This panel, if created, would provide regular scientific assessments to guide governments as they implement policies and programs related to pandemics. The scope of such a panel must include primary pandemic prevention alongside preparedness and response. The panel could be modeled after the Intergovernmental Panel on Climate Change or the Intergovernmental Platform on Biodiversity and Ecosystem Services^74,75^.

We recognize a risk of fragmentation with multiple different panels focused on climate, biodiversity, and pandemics. It is critical, therefore, to assure their coordination. By doing so, repeated efforts can be avoided, and, where applicable, intersectoral solutions can be implemented to harness co-benefits and synergies across sectors.

Moreover, there is a need to critically evaluate the evidence for the effectiveness of various pandemic prevention, preparedness, and response strategies. Although the global health community widely endorses strategies such as disease surveillance, perhaps largely due to their familiarity and experience with such methods, investments in primary prevention remain unprioritized. This raises a critical question: is there evidence that surveillance offers a greater reduction in pandemic risk compared to primary pandemic prevention (for example, is surveillance likely to activate response strategies in time to prevent spread of a pathogen with high transmissibility and pre-symptomatic spread)? To address these issues, an independent, broadly representative body could provide unbiased and politically neutral evaluation of the various strategies, encompassing prevention, preparedness, mitigation, and response^75^.

### Metrics for pandemic prevention

Any program to mitigate pandemic risk through the conservation and restoration of nature must be evaluated to ensure it has the intended impact. Thus, we propose that the Intergovernmental Panel for Pandemics develop clear and robust metrics. These metrics should not only evaluate primary pandemic prevention efforts but also integrate them into existing biodiversity and climate change frameworks. Such metrics could monitor program performance, ensure accountability and transparency, and guide equitable wealth distribution to local communities based on program outcomes.

Numerous existing biodiversity assessment metrics could be shared with pandemic prevention metrics. Examples include the Ecological Integrity Index, STAR biodiversity index, and SEED biocomplexity metric, all in line with the CBD protocols. Additionally, there needs to be metrics specifically addressing spillover risk, including the guidance presented here (e.g., protect habitats where reservoir hosts forage and rest, especially during periods of resource scarcity; and reduce land-use changes that increase human-wildlife encounters).

The development of these metrics presents an opportunity to maximize the co-benefits of biodiversity preservation, climate change mitigation, and pandemic prevention. Such an integrated and synergistic approach should increase the success of program implementation globally^75,76^. For instance, restoration of koala (*Phascolarctos cinereus*) habitats in Australia, if strategically focused on trees that both support koalas and provide nectar for bats, could concurrently restore water catchments, sequester carbon, and reduce the risk of bat virus spillovers^33^.

### Empowering local communities through One Health efforts

The One Health approach–popularized in recent years to optimize the health of people, animals, and ecosystems^77^–offers opportunities to implement ecological countermeasures for primary pandemic prevention. Currently, however, One Health efforts are overwhelmingly focused on disease surveillance in livestock and humans, rarely considering environmental drivers of emerging health threats^78^. One of the bottlenecks to advancing a more holistic One Health practice is the lack of practitioners across the animal-human-environment fields. To bridge this gap, we propose the creation of networks of ecosystem health workers to operationalize One Health and support local communities in implementing primary pandemic prevention. Those ecosystem health workers—who may include local forestry, wildlife, veterinary, medical, or public health officers–could be trained in, and help develop and implement, locally relevant ecological countermeasures, while embedded in larger governmental One Health teams. Their duties could include environmental education and ecological consultation (Supplementary Table 1), and information collection relevant to management actions (Box 3). They could also engage local universities and create pipelines for research on ecological countermeasure implementation and monitoring. They could ensure that local information is reported to national and international entities to inform effective, equitable decision-making^79^.

In parallel, it is essential to recognize the vital role of IPLCs in this framework. Integrating the perspectives and knowledge of IPLCs is not just a matter of cultural respect and justice; it is also a pragmatic strategy for designing and implementing appropriate, feasible and practical ecological countermeasures. Collaborating with IPLCs will help ensure that countermeasures align with local context and meaningfully incorporate local and Indigenous knowledge. IPLCs have managed natural ecosystems for thousands of years, and their involvement is increasingly seen as critical for reaching global climate and conservation goals^80^. Engaging IPLCs as equal partners in designing and implementing solutions to threats such as pandemics and climate change will increase the chances of successful outcomes^80,81^.

Box 3 Key questions for risk assessment and mitigation through ecological countermeasures, using bats as an example ***Natural systems focus:***

- Which species of bats are present?
- To what extent are local roost sites and foraging areas mapped?
- Are local roost sites, and buffers around these sites, protected from disturbance?
- What and where are the highest-quality habitats for these species in each season?
- What resources are limited, either seasonally or consistently?
- What habitat is required to ensure food is available during critical life stages?
- How well are the local bat biology and movement patterns understood?

***Human interactions focus:***

- Is land-use change likely to change the distribution and decrease the availability of bat foraging grounds, increase encounter rates with humans, or increase disturbance to roosts?
- What is the nature of current bat-human interactions?
- Are bat-human interactions increasing and, if so, why?
- What are the attitudes of local communities toward bats, and why?
- Who has regulatory authority to implement countermeasures?
- Who are the key stakeholders needed to develop implementation mechanisms?
- Is the available information sufficient to make informed decisions or actions?
- Can areas critical to bats’ viability and health be protected or restored?
- What steps can be taken to reduce contact between people and bats?

### Expand the evidence base for ecological countermeasures

Our current understanding of pathogen spillover is characterized by vast knowledge inequalities. Biomedical aspects of spillover are extensively explored, while ecological components of spillover are under-represented. For example, thousands of publications detail the entry of bat-origin coronaviruses into human cells, but only a few studies explore their circulation in nature^82^. Moreover, studies on spillover are relatively rare but studies that examine the entire spillover process—from environmental drivers to reservoir hosts to human infections—are exceptionally rare. Therefore, our understanding of spillover is built on partial knowledge, such as studies demonstrating increased frequency of animal-human contact following habitat loss, or higher shedding in animals under stress (Supplementary Table 2). Although there is strong evidence for these component drivers of spillover, there is a critical need for studies that encompass the entire spectrum of spillover stages, including wildlife ecology, wildlife viral dynamics, human exposure, and human infection. Such studies need to be transdisciplinary, landscape-scale, with replication in space and time, shared data, and integration of local knowledge. Critically, these investigations must be grounded in the ecological systems where pandemics are likely to originate.

Pandemics have predominantly been addressed through a biomedical lens. While biomedical approaches are an essential part of the pandemic response toolbox, the genesis of a pandemic is rooted in ecological systems, necessitating ecological approaches for prevention. By aligning our research priorities with this understanding, we can build a comprehensive set of preemptive countermeasures that mitigate pandemic risk.

## Conclusions

Spillover is an ecological process and, in the realm of human health, an ecological problem. While the human health issues arising from spillover events, such as outbreaks and pandemics, are addressed by epidemiological and biomedical countermeasures (e.g., testing, isolation, vaccines), the ecological aspects of spillover necessitate ecological solutions. In an ideal world, successful ecological countermeasures, which prevent spillover, would greatly reduce the need for biomedical countermeasures. We do not live in an ideal world; thus, we must move forward on both fronts.

To date, biomedical countermeasures to treat pandemics have received far more attention than ecological countermeasures. Our goal here has been to highlight the use of targeted ecological interventions as sensible, equitable, and efficient methods to prevent pandemics. While currently underutilized, ecological countermeasures have demonstrated potential in preventing spillover^33,76^. As challenges such as climate change, biodiversity loss, and a growing global population intensify, the relevance and necessity of ecological approaches for pandemic prevention are expected to increase.

Although we illustrate the science of ecological countermeasures using bats as a case study, the concepts are applicable across various wildlife reservoir host taxa, including ungulates, primates, and rodents. To reduce the likelihood of pandemics, we must protect where animals forage and rest so that we can keep wildlife healthy, minimize allostatic load, reduce the need for animals to alter their spatial behavior, and minimize risky human-wildlife encounters.

The current confluence of political will, resources, and scientific evidence for primary pandemic prevention provides an opportunity to incorporate ecological countermeasures into multiple policy frameworks. Such countermeasures can help prevent pandemics by, in part, protecting and restoring nature across the globe. Explicit consideration of such countermeasures within global land management and conservation strategies is key to simultaneously addressing the intertwined threats of biodiversity loss, climate change and global pandemics.

### Supplementary information

Supplementary Information
