# Supplementary material for: Ecological countermeasures to prevent pathogen spillover and subsequent pandemics
Source: Nat Commun. 2024 Mar 26;15:2577. doi: 10.1038/s41467-024-46151-9 (PMC10965931; doi:10.1038/s41467-024-46151-9)
Supplement: Supplementary file 1 — Supplementary Information [file 41467_2024_46151_MOESM1_ESM.pdf]

Supplementary Information

Ecological countermeasures to prevent pathogen spillover and subsequent pandemics

Raina K. Plowright[1]*, Aliyu N. Ahmed[2], Tim Coulson[3], Thomas W. Crowther[4], Imran Ejotre[5], Christina L. Faust[6], Winifred F. Frick[7,8], Peter J. Hudson[9], Tigga Kingston[10], P.O. Nameer[11], M. Teague O'Mara[7], Alison J. Peel[12], Hugh Possingham[13], Orly Razgour[14], DeeAnn M. Reeder[15], Manuel Ruiz-Aravena[1], Nancy B. Simmons[16], Prashanth N. Srinivas[17], Gary M. Tabor[18], Iroro Tanshi[19-21], Ian G. Thompson[22], Abi T. Vanek[23,24], Neil M. Vora[25], Charley E. Willison[1], Annika T.H. Keeley[18].

Corresponding author: rkp57@cornell.edu

The PDF file includes:

Supplementary Table 1
Supplementary Table 2

Supplementary Table 1. Examples of fine-scale ecological countermeasures

Description	Key findings or goals	Location of case study	Reference
**Enhance bat health by protecting where bats eat**			
Restoring winter-flowering trees to provide food resources for flying foxes	Interactions between land-use change and climate now lead to persistent flying fox (*Pteropus alecto*) residency in agricultural areas, where periodic food shortages drive clusters of spillovers. Pulses of winter flowering of trees in remnant forests appeared to prevent spillover; replanting winter forests may reduce the risk of spillover.	Australia	Eby et al. 2023[1]
Integrated community level plan for managing human-bat interactions and protecting where bats feed and roost	The purpose of the plan is to provide a framework to reduce impacts of flying foxes on people, whilst conserving flying foxes and the ecosystem services they provide. Effective community action informed by science and consistent with high level plans.	Australia	Eurobodalla Shire Council 2018[2]
Retaining or planting native trees and shrubs amongst crops (agroforestry)	Diverse cacao and banana agroforestry systems contribute to conservation efforts by serving as habitats to high numbers of bird and bat species.<br><br>Agroecosystems value for conservation of fruit and nectar-eating bats increases as the number of fruit-bearing trees increases.	Mexico, South America, Tanzania	Harvey & Villalobos 2007[3]<br><br>Castro-Luna & Galindo-González 2012[4]<br><br>Helbig-Bonitz et al. 2015[5]<br><br>Williams-Guillén & Perfecto 2010[6]

	Some habitat and feeding guilds, in particular forest specialist and frugivorous birds, were highly sensitive to land-use intensification. Total bat and bird abundance was mediated primarily by the availability of the respective food resources.<br><br>Less intensively managed coffee agroforests can serve as valuable feeding and commuting areas for most leaf-nosed bats (Phyllostomidae). Maintaining forest fragments in agricultural landscapes contributes to bat diversity.<br><br>Diverse shade coffee plantations serve as valuable foraging and commuting habitat for aerial insectivorous bats; several species also commute through or forage in low-shade coffee monocultures.<br><br>Great fruit-eating bats *Artibeus lituratus* captured in 'silvopastoral' areas that used agroforestry, along with no chemicals, had higher body weights and body condition scores than those within conventional farming areas.		Williams-Guillén & Perfecto 2011[7]<br><br>Chacón-Pacheco & Ballesteros-Correa 2019[8]
Empowering and incentivizing local communities	Local stewardship improves conservation outcomes.	Uganda	Nakakaawa et al. 2015[9]<br><br>Ejotre et al. 2022[10]
Allocating fragile habitats for community	Local protection of sensitive habitats, to reduce encroachment of	Uganda	Ejotre et al. 2022[10]

beekeeping and group custody	a community forest in West Nile, Uganda.		
Agave plant restoration near bat roosts in a migratory corridor	Restoring foraging resources near roosts.	Mexico and Southwestern USA	Bat Conservation International[11]
Reducing wildfire outbreaks in forests and other native habitat	Study ongoing	Nigeria	Iroro Tanshi, personal communication[12]
Reducing forest logging through anti-logging patrols	Study ongoing	Nigeria	Iroro Tanshi, personal communication[12]
Creating systems of connected protected areas	The document provides guidance on creating well-connected systems of protected and conserved areas that support a diversity of ecological functions including disease control, migration, water and nutrient cycling, pollination, seed dispersal, food security, and climate resilience.		Hilty et al. 2020[13]
Maintain and restore connectivity in farmland and urban areas	Study investigated the impact of connectivity and configuration of structural landscape elements on flight activity, species richness and diversity of insectivorous bats. The authors recommend that the reintroduction of structural elements to increase habitat heterogeneity should become part of agri-environment schemes.	Switzerland	Frey-Ehrenbold et al. 2013[14]
**Enhance bat health by protecting where bats roost**			
Concurrent community education and	Minimizing conflict at the human-bat interface while safeguarding	Uganda, Kenya	Ejotre et al. 2022[10]

conservation actions on bats roosting in or close to human spaces	established bat roosts within human spaces.<br><br>Deployed during the Covid-19 pandemic to save study colonies.		Webala, Musila & Makau 2014[15]
Preventing or reducing hunting and consumption of bats	Study ongoing	Nigeria	Iroro Tanshi, personal communication.[12,16]
Imposing restrictions on cave visitation	A management plan was created that accounted for seasonality of the bat community and how the bats used the cave (hibernation vs nursing). Plans allowed managers to minimize contact with the bats and reduce disturbance at critical times of year.<br><br>Community imposed/controlled quarters for guano harvesting in caves in northern Rwanda and south coast of Kenya.	Turkey, Kenya, Rwanda	Paksuz & Özkan 2012[17]<br><br>Paul W. Webala, personal communication[18]
Working with farmers, local communities, and pest controllers to reduce indiscriminate killing of bats	In this study, men were more likely to intend to indiscriminately kill bats if they knew less about bat natural history and/or had previously suffered vampire bats attacking their livestock. Men knew more about bat natural history and were less likely to harbor indiscriminate bat-killing intentions if they had experienced some form of environmental education.	Costa Rica	Reid 2016[19]<br><br>Levers4Health[20]
Using non-lethal measures to prevent bats from accessing fruit in orchards	Several effective options for reducing bat-human interactions, these include netting, decoy crops,	Australasia, Africa and Oceania	Aziz et al. 2016[21]

to reduce human-wildlife conflict	bamboo skirts around sap, noise and smell deterents.		
Public education and community outreach at roosts, including leveraging controlled roost (cave, tree) tourism and community protection of the roosts	Ensure long-term protection of cave roosts and of straw-colored fruit bat (*Eidolon helvum)* tree roosts in public spaces and busy villages	Kenya	Ongoing activities by Paul W. Webala and students at cave roosts at the Kenyan coast and at several *Eidolon* roosts around the country: Webala, Musila & Makau 2014[15]
Following the guidelines for minimizing the negative impact to bats and other cave organisms from guano harvesting	The document provides guidance on planning, implementing, and monitoring guano harvesting operations with consideration for the overall well-being of caves, bats, and cave invertebrates, and to support continued guano harvests.		IUCN SSC 2014[22]
Creation of Nakanaca Cave Preserve on Vanua Levu, Fiji, to protect the Fijian Free-tailed Bat (*Chaerephon bregullae*) in collaboration with local village and NGOs	Reduced human entry and consumption of bats.	Fiji	Jon Flanders, Bat Conservation International, personal communication[23]
Protection of cave roosts of critically endangered bats	Works is in progress to fence perimeters to reduce guano harvesting and reduce human entry into bat caves. Stony Hill Cave was purchased and transferred for management by Jamaican National Environmental Protection Agency (NEPA) in 2021 by Bat Conservation International.	Jamaica	Jon Flanders, personal communication[23]

Building bat boxes to conserve bats	Increase in local bat populations and reduction in mosquito populations (reduced human malaria cases).	USA	Levers4Health[24]
Cave patrols	Deter bat hunting	Nigeria	Iroro Tanshi, personal communication[12]
Cave hunting bans	Community consensus facilitated policy to reduce hunting in caves.	Nigeria	Iroro Tanshi, personal communication[12]
Alternative protein sources and livelihoods.	Community consensus to discourage dependence on bat meat.	Nigeria	Iroro Tanshi, personal communication[12]
**Protect people and livestock at risk**			
Bamboo skirt barriers around date palm sap	Prevent contamination of date palm sap with bat urine, thus reducing Nipah virus transmission.<br><br>A Randomized Controlled Trial of Interventions to Impede Date Palm Sap Contamination by Bats to Prevent Nipah Virus Transmission in Bangladesh.	Bangladesh	Nahar et al. 2014[25]<br><br>Khan et al. 2012[26]<br><br>Levers4Health[27]
Ultrasound deterrent	Human deterrence of bats to reduce activity in certain areas.		Gilmour et al. 2020[28]
Drone deterrents	Deterrence of bats to reduce conflict with human-made structures.		Werber et al. 2020[29]
**Government and community laws/policies**			
Collaborative natural resource management	Strengthens local level governance of natural resources.	Uganda	Kazoora et al. 2020[30]

Laws prohibiting wildlife trade	Wildlife hunting was made illegal and sale of wildlife products was prohibited.<br><br>Conservation of general wildlife populations	The Gambia	The Gambia Biodiversity Act 2003[31]
Individual limitations on tree harvesting	Individuals in certain communities are entitled to cut down only one tree in their lifetime and must plant 10 trees as replacement.<br><br>Conservation of biodiversity at landscape scales.	The Gambia	The Gambia Biodiversity Act 2003[31]
Establishment of protected areas	Conservation of biodiversity at landscape scales.	The Gambia	The Gambia Biodiversity Act 2003[31]
Use of appropriate community communication channels to create awareness	For example, Kyengilan cultural dance group in Gambia that delivers message through their performance.<br><br>Facilitates environmental protection (not exclusive for bats but relevant to all biodiversity).	The Gambia	The Gambia Biodiversity Act 2003 [31]

Supplementary Table 2. Examples of studies on components of the spillover process

Component of Spillover	Mechanism	Study type	Geographic scope	Host species (or taxa)	Pathogen (if applicable)	Response measured and direction	Proposed mechanism of action	Reference
Exposure to novel host	Increased abundance of competent recipient hosts	Cross-sectional (multiple sites)	Landscape (multiple sites within a country)	Domestic pigs	Nipah virus	Human cases of spillover in relation to pig farming (modelling).	In Malaysia, large-scale swine production facilities near mango orchards where fruit bats roost believed to be a driver of Nipah virus	Pulliam et al. 2012[32]

							transmission from fruit bat reservoirs to pigs, and eventual spillover into humans.	
Exposure to novel host	Increased conflict with wildlife	Cross-sectional (multiple sites)	Local (single site)	Humans and non-human primates (NHPs)	None	Behavioral surveys of humans for reporting contacts with NHPs; found a statistically significant association between greater forest fragmentation and higher human & NHP contact.	Increase in edge density makes forest habitat more accessible to people, increasing the potential for overlap of humans and NHPs.	Bloomfield et al. 2020[33]
Exposure to novel host	Increased movement and connectivity	Meta-analysis	Global	All	None	Increased human disturbance increases nocturnality of many diurnal mammals across the world.	Decreased temporal overlap of humans and wildlife, but increased temporal overlap among wildlife.	Gaynor et al. 2018[34]
Exposure to novel host	Increased movement and connectivity	Meta-analysis	Global	Mammals	None	Increased human footprint index decreases mammal median displacement and long-distance displacement.	Increase fragmentation, decreased habitat permeability for movement.	Tucker et al. 2018[35]
Exposure to novel host	More intensive space use	Cross-sectional (multiple sites)	Local (single site)	Egyptian fruit bat *(Rousettus aegyptiacus)*	none	Risk taking behaviors were more prevalent in urban pups compared to pups from rural locations.	Rural lactating females have lower milk cortisol than those in urban areas, and these levels can be linked to behaviors.	Harten et al. 2021 [36]
Exposure to novel host	More intensive space use	Cross-sectional	Local (single site)	Bats	none	Bat activity (measured with sound recorders) is	More human activity on weekends drives bats away.	Li et al. 2020 [37]

		(multiple sites)				lower in urban parks on the weekends compared to the week.		
Exposure to novel host	More intensive space use	Observational (single site)	Local (single site)	Solitary fruit-eating bat *(Dermanura watsoni)*	none	Larger daily ranges were associated with higher use of degraded habitats.	Home range geometry and composition of focal foraging areas indicated that wider ranging bats performed directional foraging bouts from natural to degraded forest sites traversing the matrix.	Ripperger et al. 2015 [38]
Immune, molecular, and structural barriers to infection in novel host	Increased susceptibility to disease	Cross-sectional (multiple sites)	Local (single site)	Mexican free-tailed bat *(Tadarida brasiliensis)*	bacteria	No systemic differences in bacterial killing assay (BKA) and phytohaemagglutin assay (PHA) results between cave and bridge bats.	BKA is a measure of overall innate response. PHA is a measure of T cell-mediated response.	Allen et al. 2009 [39]
Immune, molecular, and structural barriers to infection in novel host	Increased susceptibility to disease	Cross-sectional (multiple sites)	Local (single site)	Mexican free-tailed bat *(Tadarida brasiliensis)*	none	No difference in glucosteroids (GC) between bridge and cave habitats.	GC levels should increase from baseline during stress, but no difference was detected.	Allen et al. 2011 [40]
Immune, molecular, and structural barriers to infection in novel host	Increased susceptibility to disease	Cross-sectional (multiple sites)	Local (single site)	Diadem leaf-nosed bat *(Hipposideros diadema)*	None	Immune function, measured by neutrophil-to-lymphocyte (N:L) ratio, varied with cave disturbance by humans but not cave complexity. N:L ratio was	An increase in N:L ratio is indicative of overall physiological stress and/or allostatic load. However, N:L ratio may decrease here due to acclimatization to chronic stress. Alternately, higher landscape disturbance	Phelps & Kingston 2018 [41]

						lowest in most-disturbed caves.	may increase total pathogen pressure and alter subsequent leukocyte response.	
Immune, molecular, and structural barriers to infection in novel host	Increased susceptibility to disease	Cross-sectional (multiple sites)	Landscape (multiple sites within a country)	Flat-faced fruit-eating bat *(Artibeus planirostris),* Seba's short-tailed bat *(Carollia perspicillata),* Parnell's mustached bat *(Pteronotus parnelli)*	Bat flies	Bat fly abundance increased and decreased with higher human populations.	Increased human population disturbance affects bat flies.	Pilosof et al. 2012 [42]
Immune, molecular, and structural barriers to infection in novel host	Increased susceptibility to disease	Cross-sectional (multiple sites)	Local (single site)	Bornean horseshoe bat *(Rhinolophus borneensis)*	None	Cave-roosting bats from fragmented habitats had higher N:L ratios compared to conspecifics from active logging areas. Habitat had no effect on body mass.	Increased edge habitat due to fragmentation may increase food availability for *Rhinolophus spp.*	Seltmann et al. 2017 [43]
Immune, molecular, and structural barriers to infection in novel host	Increased susceptibility to disease	Cross-sectional (multiple sites)	Local (single site)	Lesser woolly horseshoe bat *(Rhinolophus sedulus),* Trefoil horseshoe bat *(R. trifoliatus)*	None	Actively logged & fragmented habitats decrease body mass and total WBC in foliage-roosting bat species. No effect on N:L ratio.	Increased edge habitat with fragmentation may increase food availability for *Rhinolophus spp.*	Seltmann et al. 2017 [43]
Pathogen prevalence, infection intensity and shedding from hosts	Change in nutritional plane (+/-)	Experimental	Local (single site)	American robin (*Turdus migratorius*)	West Nile Virus	Birds with restricted diets had higher viral loads in blood and were infected for longer, making	Low food resources prevented immune system from mounting initial and sustained neutralizing response.	Owen et al. 2021 [44]

						transmission to other birds more efficient.		
Pathogen prevalence, infection intensity and shedding from hosts	Toxic direct and indirect effects on immune function	Cross-sectional (multiple sites)	Local (single site)	Neotropical Bat Species	*Bartonella*	Bartonella infection related to individual immune cell response and mercury concentrations; no systematic difference.	Bats with higher mercury levels have higher neutrophils but not lymphocytes or Bartonella infections.	Becker et al. 2021 [45]
Reservoir host distribution, density and behavior	Attract hosts to anthropogenic areas	Cross-sectional (multiple sites)	Landscape (multiple sites within a country)	Carnivores	none	Diet overlap among carnivores increased with human activity.	Carnivores in close proximity to humans have increased trophic overlap: increased d13C and increase d15N values in diet. There is also evidence that carnivores eat more human food in more disturbed areas.	Manlick & Pauli 2020 [46]
Reservoir host distribution, density and behavior	Disturbance and culling as a result of conflict	Cross-sectional (multiple sites)	Landscape (multiple sites within a country)	Common vampire bat *(Desmodus rotundus)*	Rabies Virus	Rabies virus seropositivity increased with culling	Periodic and repeated culling associated with increases rabies virus seroprevalence	Streicker et al. 2012 [47]
Reservoir host distribution, density and behavior	Increased abundance of competent reservoir hosts	Cross-sectional (multiple sites)	Landscape (multiple sites within a country)	Multimammate mouse (*Mastomys natalensis*), and others	*Yersinia pestis* (plague)	Proportion of the rodent population with serological evidence of exposure to the pathogen (seroprevalence) was twice as high in agricultural areas compared to conserved areas.	Disturbed areas have less rodent diversity and greater proportions of species that are highly competent for transmitting *Y. pestis*, thus proportion of the reservoir population with serological evidence of exposure to the pathogen (seroprevalence) was twice as high in agricultural areas.	McCauley et al. 2015 [48]

Reservoir host distribution, density, and behavior	Increased abundance of competent reservoir hosts	Cross-sectional (multiple sites)	Landscape (multiple sites within a country)	Multiple rodents: Northern pygmy rice rat (*Oligoryzomys fulvescens)*, Mexican harvest mouse (*Reithrodontomys mexicanus)*, Western harvest mouse (*Reithrodontomys megalotis)*	Hantavirus	Seroprevalence of Hantavirus is higher in disturbed landscapes and remnant forest edges near urban areas.	Rodents that are competent hosts of Hantavirus become more dominant in disturbed landscapes and edges near urban areas, leading to higher seroprevalence of hantavirus.	Suzán et al. 2009, 2008 [49,50]
Reservoir host distribution, density, and behavior	Increased abundance of competent reservoir hosts	Cross-sectional (multiple sites)	Global	384 host species (strongest effect: rodents, passerines, bats)	zoonotic vs. non-zoonotic	Hosts of zoonotic pathogens were more species-rich and abundant in disturbed environments (secondary forests, agricultural land, urban areas) compared to pristine habitat.	Argue that ecological or life-history traits that influence host status (harboring zoonoses) are also linked to tolerance to human disturbance.	Gibbs et al. 2021 [51]
Reservoir host distribution, density, and behavior	More aggregation around resources	Cross-sectional (multiple sites)	Local (single site)	Deer mouse (*Peromyscus maniculatus)* (reservoir), Desert woodrat (*Neotoma lepida)*	Sin Nombre virus	Seroprevalence of Sin Nombre virus in different sites.	Human disturbance increases local population density of rodents, led to longer travelling distances of rodents, and reduced rodent population immunocompetence.	Mackelprang et al. 2001 [52]
Reservoir host distribution, density, and behavior	More aggregation around resources	Cross-sectional (multiple sites)	Local (single site)	Tent-making bat *(Uroderma bilobatum)*	Tent-making bat hepatitis B virus (TBHBV)	Bats in areas with lower forest cover have higher prevalence of TBHBV.	Females more infected than males, especially during pregnancy.	Hiller et al. 2018 [53]
Reservoir host	More aggregation	Cross-sectional	Local (single site)	Jamaican fruit bat	Bat flies	Prevalence and intensity of bat fly	Fragmented habitat with fewer potential roosting	Hiller et al. 2020 [54]

distribution, density, and behavior	around resources	(multiple sites)		*(Artibeus jamaicensis)*		infestation in response to habitat change - found multiple relationships.	sites leads to increased parasite prevalence.	
Reservoir host distribution, density, and behavior	More aggregation around resources	Cross-sectional (multiple sites)	Landscape (multiple sites within a country)	Bats	None	Species diversity across a landscape.	Smaller fragments, edges and regrowth sites had fewer bat species and higher levels of dominance than contiguous forests.	Rocha et al. 2017 [55]
Reservoir host distribution, density, and behavior		Meta-analysis	Global (five countries)	Small mammals	Hantavirus	Hantavirus reservoir species richness or abundance increases in fragmented habitats.	Loss in overall species richness facilitated increase in abundance of hantavirus reservoir species.	Rubio et al. 2014 [56]

References

1. Eby, P. *et al.* Pathogen spillover driven by rapid changes in bat ecology. *Nature* **613**, 340–344 (2022).
2. Eurobodalla Shire Council. Eurobodalla Flying-fox Management Plan. (2018).
3. Harvey, C. A. & González Villalobos, J. A. Agroforestry systems conserve species-rich but modified assemblages of tropical birds and bats. *Biodivers. Conserv.* **16**, 2257–2292 (2007).
4. Castro-Luna, A. A. & Galindo-González, J. Enriching agroecosystems with fruit-producing tree species favors the abundance and richness of frugivorous and nectarivorous bats in Veracruz, Mexico. *Mamm. Biol.* **77**, 32–40 (2012).
5. Helbig-Bonitz, M. *et al.* Bats are Not Birds – Different Responses to Human Land-use on a Tropical Mountain. *Biotropica* **47**, 497–508 (2015).

6. Williams-Guillén, K. & Perfecto, I. Effects of Agricultural Intensification on the Assemblage of Leaf-Nosed Bats (Phyllostomidae) in a Coffee Landscape in Chiapas, Mexico. *Biotropica* **42**, 605–613 (2010).
7. Williams-Guillén, K. & Perfecto, I. Ensemble Composition and Activity Levels of Insectivorous Bats in Response to Management Intensification in Coffee Agroforestry Systems. *PLOS ONE* **6**, e16502 (2011).
8. Chacon-Pacheco, J. J. & Ballesteros-Correa, J. Mejor condición corporal de Artibeus lituratus en fragmentos de bosque seco asociados a sistemas silvopastoriles que en sistemas convencionales de ganadería en Córdoba, Colombia. *Oecologia Aust.* **23**, 589–605 (2019).
9. Nakakaawa, C., Moll, R., Vedeld, P., Sjaastad, E. & Cavanagh, J. Collaborative resource management and rural livelihoods around protected areas: A case study of Mount Elgon National Park, Uganda. *For. Policy Econ.* **57**, 1–11 (2015).
10. Ejotre, I., Reeder, D. M., Matuschewski, K., Kityo, R. & Schaer, J. Negative Perception of Bats, Exacerbated by the SARS-CoV-2 Pandemic, May Hinder Bat Conservation in Northern Uganda. *Sustainability* **14**, 16924 (2022).
11. Bat Conservation International. Restoring Agave for Nectar-feeding bats. *Bat Conservation International* https://www.batcon.org/our-work/protect-restore-landscapes/agave-restoration/ (2023).
12. Plowright, R. K. Personal Communication with Iroro Tanshi. (2022).
13. Hilty, J. *et al. Guidelines for conserving connectivity through ecological networks and corridors*. (IUCN, 2020). doi:10.2305/IUCN.CH.2020.PAG.30.en.

14. Frey-Ehrenbold, A., Bontadina, F., Arlettaz, R. & Obrist, M. K. Landscape connectivity, habitat structure and activity of bat guilds in farmland-dominated matrices. *J. Appl. Ecol.* **50**, 252–261 (2013).
15. Webala, P. W., Musila, S. & Makau, R. Roost Occupancy, Roost Site Selection and Diet of Straw-Coloured Fruit Bats (Pteropodidae: Eidolon helvum) in Western Kenya: The Need for Continued Public Education. *Acta Chiropterologica* **16**, 85–94 (2014).
16. Small Mammal Conservation Organization. Community action to save the rare Endangered Short-tailed Roundleaf bat. (2023).
17. Paksuz, S. & Özkan, B. The protection of the bat community in the Dupnisa Cave System, Turkey, following opening for tourism. *Oryx* **46**, 130–136 (2012).
18. Plowright, R. K. Personal Communication with Paul W. Webala. (2022).
19. Reid, J. L. Knowledge and Experience Predict Indiscriminate Bat-Killing Intentions among Costa Rican Men. *Biotropica* **48**, 394–404 (2016).
20. Levers4Health. Educate people about living safely with bats to reduce spillover and conserve bats. *Levers4Health* https://jvr782.wixsite.com/leversforhealth/bat-education-solution (2021).
21. Aziz, S. A., Olival, K. J., Bumrungsri, S., Richards, G. C. & Racey, P. A. The Conflict Between Pteropodid Bats and Fruit Growers: Species, Legislation and Mitigation. in *Bats in the Anthropocene: Conservation of Bats in a Changing World* (eds. Voigt, C. C. & Kingston, T.) 377–426 (Springer International Publishing, 2016). doi:10.1007/978-3-319-25220-9_13.

22. International Union for Conservation of Nature (IUCN). *IUCN SSC Guidelines for Minimizing the Negative Impact to Bats and Other Cave Organisms from Guano Harvesting*. 1–13 https://portals.iucn.org/library/sites/library/files/documents/Rep-2014-002.pdf (2014).
23. Plowright, R. K. Personal Communication with Jon Flanders (Bat Conservation International). (2022).
24. Levers4Health. Build bat boxes to conserve bats and reduce human malaria. *Levers4Health* https://jvr782.wixsite.com/leversforhealth/bat-box-solution (2021).
25. Nahar, N. *et al.* Raw Sap Consumption Habits and Its Association with Knowledge of Nipah Virus in Two Endemic Districts in Bangladesh. *PLOS ONE* **10**, e0142292 (2015).
26. Khan, S. U. *et al.* A randomized controlled trial of interventions to impede date palm sap contamination by bats to prevent nipah virus transmission in Bangladesh. *PLOS ONE* **7**, e42689 (2012).
27. Levers4Health. Use bamboo skirts on sap pots to reduce Nipah virus spillover and conserve bats. *Levers4Health* https://jvr782.wixsite.com/leversforhealth/bamboo-skirts-solution (2021).
28. Gilmour, L. R. V., Holderied, M. W., Pickering, S. P. C. & Jones, G. Comparing acoustic and radar deterrence methods as mitigation measures to reduce human-bat impacts and conservation conflicts. *PLOS ONE* **15**, e0228668 (2020).
29. Werber, Y., Hareli, G., Yinon, O., Sapir, N. & Yovel, Y. Drone-mounted audio-visual deterrence of bats: implications for reducing aerial wildlife mortality by wind turbines. *Remote Sens. Ecol. Conserv.* **n/a**,.

30. Kazoora, C., Irumba, D., Smith, N. & Campese, J. *A Review of Collaborative Forest Management in Uganda*. 1–40 https://www.nfa.go.ug/images/A_REVIEW_OF_COLLABORATIVE_FOREST_MANAGEMENT_IN_UGANDA.pdf (2020).
31. UNEP Law and Environment Assistance Platform. Biodiversity and Wildlife Act. *Biodiversity and Wildlife Act 2003 (Gambia)* https://leap.unep.org/countries/gm/national-legislation/biodiversity-and-wildlife-act (2003).
32. Pulliam, J. R. C. *et al.* Agricultural intensification, priming for persistence and the emergence of Nipah virus: a lethal bat-borne zoonosis. *J. R. Soc. Interface* **9**, 89–101 (2012).
33. Bloomfield, L. S. P., McIntosh, T. L. & Lambin, E. F. Habitat fragmentation, livelihood behaviors, and contact between people and nonhuman primates in Africa. *Landsc. Ecol.* **35**, 985–1000 (2020).
34. Gaynor, K. M., Hojnowski, C. E., Carter, N. H. & Brashares, J. S. The influence of human disturbance on wildlife nocturnality. *Science* **360**, 1232–1235 (2018).
35. Tucker, M. A. *et al.* Moving in the Anthropocene: Global reductions in terrestrial mammalian movements. *Science* **359**, 466–469 (2018).
36. Harten, L. *et al.* Urban bat pups take after their mothers and are bolder and faster learners than rural pups. *BMC Biol.* **19**, 190 (2021).
37. Li, H. *et al.* The Weekend Effect on Urban Bat Activity Suggests Fine Scale Human-Induced Bat Movements. *Animals* **10**, 1636 (2020).

38. Ripperger, S. P., Kalko, E. K. V., Rodríguez-Herrera, B., Mayer, F. & Tschapka, M. Frugivorous Bats Maintain Functional Habitat Connectivity in Agricultural Landscapes but Rely Strongly on Natural Forest Fragments. *PLOS ONE* **10**, e0120535 (2015).

39. Allen, L. C. *et al.* Roosting ecology and variation in adaptive and innate immune system function in the Brazilian free-tailed bat (Tadarida brasiliensis). *J. Comp. Physiol. [B]* **179**, 315 (2009).

40. Allen, L. C. *et al.* Variation in Physiological Stress between Bridge- and Cave-Roosting Brazilian Free-Tailed Bats. *Conserv. Biol.* **25**, 374–381 (2011).

41. Phelps, K. L. & Kingston, T. Environmental and biological context modulates the physiological stress response of bats to human disturbance. *Oecologia* **188**, 41–52 (2018).

42. Pilosof, S., Dick, C. W., Korine, C., Patterson, B. D. & Krasnov, B. R. Effects of Anthropogenic Disturbance and Climate on Patterns of Bat Fly Parasitism. *PLOS ONE* **7**, e41487 (2012).

43. Seltmann, A. *et al.* Habitat disturbance results in chronic stress and impaired health status in forest-dwelling paleotropical bats. *Conserv. Physiol.* **5**, (2017).

44. Owen, J. C. *et al.* Reservoir hosts experiencing food stress alter transmission dynamics for a zoonotic pathogen. *Proc. R. Soc. B Biol. Sci.* **288**, 20210881 (2021).

45. Becker, D. J. *et al.* Disentangling interactions among mercury, immunity and infection in a Neotropical bat community. *J. Appl. Ecol.* **58**, 879–889 (2021).

46. Manlick, P. J. & Pauli, J. N. Human disturbance increases trophic niche overlap in terrestrial carnivore communities. *Proc. Natl. Acad. Sci.* **117**, 26842–26848 (2020).

47. Streicker, D. G. *et al.* Ecological and anthropogenic drivers of rabies exposure in vampire bats: implications for transmission and control. *Proc. R. Soc. B Biol. Sci.* **279**, 3384–3392 (2012).

48. McCauley, D. J. *et al.* Effects of Land Use on Plague (Yersinia pestis) Activity in Rodents in Tanzania. *Am. J. Trop. Med. Hyg.* **92**, 776–783 (2015).

49. Suzán, G. *et al.* The Effect of Habitat Fragmentation and Species Diversity Loss on Hantavirus Prevalence in Panama. *Ann. N. Y. Acad. Sci.* **1149**, 80–83 (2008).

50. Suzán, G. *et al.* Experimental Evidence for Reduced Rodent Diversity Causing Increased Hantavirus Prevalence. *PLoS ONE* **4**, e5461 (2009).

51. Gibb, R. *et al.* Zoonotic host diversity increases in human-dominated ecosystems. *Nature* **584**, 398–402 (2020).

52. Mackelprang, R., Dearing, M. D. & St Jeor, S. High prevalence of Sin Nombre virus in rodent populations, central Utah: a consequence of human disturbance? *Emerg. Infect. Dis.* **7**, 480–482 (2001).

53. Hiller, T. *et al.* Host Biology and Anthropogenic Factors Affect Hepadnavirus Infection in a Neotropical Bat. *Ecohealth* **16**, 82–94 (2019).

54. Hiller, T., Brändel, S. D., Honner, B., Page, R. A. & Tschapka, M. Parasitization of bats by bat flies (Streblidae) in fragmented habitats. *Biotropica* **52**, 488–501 (2020).

55. Rocha, R. *et al.* Consequences of a large-scale fragmentation experiment for Neotropical bats: disentangling the relative importance of local and landscape-scale effects. *Landsc. Ecol.* **32**, 31–45 (2017).

56. Rubio, A. V., Ávila-Flores, R. & Suzán, G. Responses of small mammals to habitat fragmentation: epidemiological considerations for rodent-borne hantaviruses in the Americas. *EcoHealth* **11**, 526–533 (2014).
